# Hearing children of Deaf parents: Gender and birth order in the delegation of the interpreter role in culturally Deaf families

**DOI:** 10.4102/ajod.v7i0.365

**Published:** 2018-04-30

**Authors:** Nomfundo F. Moroe, Victor de Andrade

**Affiliations:** 1 Department of Speech Pathology and Audiology, School of Human and Community Development, University of the Witwatersrand, South Africa

## Abstract

**Background:**

Culturally, hearing children born to Deaf parents may have to mediate two different positions within the hearing and Deaf cultures. However, there appears to be little written about the experiences of hearing children born to Deaf parents in the South African context.

**Objective:**

This study sought to investigate the roles of children of Deaf adults (CODAs) as interpreters in Deaf-parented families, more specifically, the influence of gender and birth order in language brokering.

**Method:**

Two male and eight female participants between the ages of 21 and 40 years were recruited through purposive and snowball sampling strategies. A qualitative design was employed and data were collected using a semi-structured, open-ended interview format. Themes which emerged were analysed using thematic analysis.

**Results:**

The findings indicated that there was no formal assignment of the interpreter role; however, female children tended to assume the role of interpreter more often than the male children. Also, it appeared as though the older children shifted the responsibility for interpreting to younger siblings. The participants in this study indicated that they interpreted in situations where they felt they were not developmentally or emotionally ready, or in situations which they felt were better suited for older siblings or for siblings of another gender.

**Conclusion:**

This study highlights a need for the formalisation of interpreting services for Deaf people in South Africa in the form of professional interpreters rather than the reliance on hearing children as interpreters in order to mediate between Deaf and hearing cultures.

## Background

Globally, it is suggested that 90% of people with audiological deafness who have children have hearing children (Christodoulou et al. 2009). International studies conducted on hearing children of deaf parents show that these children are raised in families where there appears to be unique dynamics in relation to hearing children born to hearing parents. Authors such as Preston (1995) report that hearing children of Deaf parents are raised in unique, extraordinary family settings as they may be exposed to and interact with two differing cultural, social and linguistic systems: one of their Deaf parents and the Deaf community and one of hearing peers and adults. What makes these family settings unique is the fact that cultures differ in a sense that the Deaf community uses Sign Language as a mode of communication, whereas the hearing community uses spoken language to communicate. Therefore, the lives of hearing children of Deaf adults (CODAs) may inherently incorporate the ambiguity of being culturally ‘Deaf’ and yet functionally hearing. As a result, these families, more specifically the CODAs, may then need to bridge the gap between the hearing and Deaf worlds and, therefore, may face unique communication and cultural challenges (Clark 2003). In general, there is very limited research on the experiences of CODAs in South Africa. Therefore, this study seeks to capture and highlight the experiences of hearing children born to Deaf parents in South Africa. More specifically, this study aims to describe the delegation of the language broker role in Deaf-parented families and to understand the dynamics that gender and birth order may play in the delegation of this role.

### ‘Deaf’ versus ‘deaf’

A distinction is made between audiological deafness (‘deaf’) and cultural deafness (‘Deaf’). The term ‘deaf’ refers to an audiological status, while ‘Deaf’ refers to a cultural identity (Lane, Hoffmeister & Bahan 1996; Lucas & Valli 1990). The uppercase ‘D’ in ‘Deaf’ culture signifies cultural membership in a community with a shared language and experience (Murray, Klinger & McKinnon 2007). On the other hand, the lowercase ‘d’ in ‘deafness’ is a term that refers to an audiological concept relating to hearing difficulties (Murray et al. 2007)

As a response to what Deaf persons perceive as oppressive attitudes conveyed by hearing society, members of the Deaf community have preferred to write ‘Deaf’ with a capital D instead of a lowercase letter. ‘Deaf’ signifies a person who places pride in themselves being identified as a ‘Deaf person’, a person who aligns himself or herself with Deaf Culture and Sign Language, and is accepted by the Deaf community as a Deaf person. On the other hand, the use of the term ‘deaf’ refers to the audiological dimension of the physical loss of a person’s hearing. An individual who identifies himself or herself as ‘deaf’ is considered by the Deaf community to be an ‘outsider’ as he or she does not share the same language or culture as the Deaf persons (McIlroy 2008:42). It is therefore important to note that not all audiologically deaf people belong to Deaf culture, and hence the distinction between ‘deaf’ and ‘Deaf’ (Siple 1994). According to Filer and Filer (2000), in order to begin understanding the experience of hearing children of Deaf parents, it is necessary to have a basic understanding of Deaf culture. The next section briefly discusses some tenets of Deaf culture.

### Deaf culture and the Deaf community

Similar to any culture, language is an important part of Deaf people’s identity (Clark 2003). Although not all Deaf people use Sign Language, it is still considered the single most important element that connects and binds the Deaf community together (Filer & Filer 2000). Resultantly, in the United States of America, Deaf people created a community known as DEAF-WORLD, which has its own language and culture; a community that is based on ‘shared experiences of a particular human experience, that of Deafness’, despite the fact that they are viewed as a minority group (Singleton & Tittle 2000:222). However, even though it seems exclusionary to the people outside of the Deaf community, there are often specific criteria for inclusion in the Deaf community:

Singleton and Tittle (2000) state that one is either born into the Deaf community or ‘one opts’ in when one realizes that despite one’s efforts and those of one’s hearing family, one simply cannot identify with the hearing world. (p. 222)

According to Cokely (1980), as cited by Napier (2002:142), there are four spheres of life through which people can be members of the Deaf community: through their audiological status, political support of the goals of the Deaf community, social contact within the community and through linguistic fluency in the Sign Language of the community.

Furthermore, Singleton and Tittle (2000) note that in as much as these are the core prerequisites for entry into the Deaf community, there is diversity in the membership, as this community may also include a range of people regardless of the degree of their physiological and measurable deafness. Hearing people who identify with Deaf culture, such as hearing children of Deaf parents, may also form part of the Deaf community. Because of such diversity within the community, in order for one to gain entry into the Deaf community, ‘one must adopt a cultural view of Deafness and be proficient in Sign Language’ (Singleton and Tittle 2000:222). According to Selzer (2010), the South African Deaf community is relatively small and tends to keep to itself and appears very guarded about its culture and language. Furthermore, very little is known about South African Deaf culture (Selzer 2010). Acceptance and acculturation into the Deaf community seem to be affirmed by one’s attitude and the use of Sign Language and not upon one’s audiometric status (Singleton & Tittle 2000). In addition to satisfying the criteria for membership into the Deaf community, one may still need to possess the right ‘attitude toward Deaf people, their language, culture, and minority status to be accepted into the community’ (Napier 2002:142).

### Deafness as a disability

The hearing community is reported to generally view deafness as a disability and has little understanding or information about Deaf culture (Filer & Filer 2000). In addition, Hoffmeister (1996) states that many of the professionals involved in educating Deaf people have viewed deafness as pathological by focusing on the physiological deafness. This disability perspective ought not to be seen as the authors’ perspective of Deafness but is presented to provide an in-depth understanding of the position of Deaf people and the position of CODAs in relation to the two worlds which they often inhabit and through which they have to navigate, especially considering the developing context within which this study was conducted. Within this disability perspective, ‘deafness is considered to be one of the single largest prevailing disabilities in South Africa’, eliciting growing local, national and international concerns (Storbeck 2010:502). DeafSA, the Deaf Federation of South Africa, estimates that about 10% of the South African population are disabled in some way and that approximately 3.5% have some degree of deafness. Furthermore, people with disabilities in South Africa generally lack access to or knowledge of basic health and social services (Barratt 2007), which may be attributed to the history of ‘apartheid’ (Baker 2011), where the Deaf community was often excluded from participation in the wider hearing society. Because of these limitations, the onus seems to fall on hearing children to act as the communication link and language brokers between their Deaf parents and the hearing community.

The disability view of deafness is in stark contrast to the view of the Deaf community where members consider themselves ‘neither isolated nor disabled, but rather a cultural and linguistic minority, disadvantaged by a language barrier rather than by a disability’ (Murray et al. 2007:172). Preston (1995) argues that ‘Deafness is a particular human condition understood by outsiders as a profoundly devastating disability, by insiders as an incidental feature and cultural norm’ (p. 1462). Also, Power and Leigh (2003:40) further elucidate that deafness is primarily ‘a communication disability’. Deaf people would not be regarded as disabled if they were given access to information and the means to communicate with the hearing community (Napier 2002).

### Children of Deaf Adults

The term ‘CODA’ refers to any hearing person born to one or two Deaf parents (Bishop & Hicks 2005; Bull 1998; Mand et al. 2009). Being a CODA means that there is a cultural and linguistic difference between Deaf children born to hearing parents and hearing children born to hearing parents (Bull 1998). According to Bishop and Hicks (2005), the term ‘CODA’ is reserved for people who see themselves ‘as not quite fitting into the Deaf/hearing categories; people who want to carve out a third niche for themselves’ (p. 192). It should be noted that a hearing child born to one Deaf parent and one hearing parent is still referred to as a CODA. Quigley and Paul (1990) estimate that approximately 5% of CODAs are born to two Deaf parents and 10% of CODAs are born to one Deaf parent and one hearing parent. Mallory, Schein, and Zingle (1992) state that 10% of Deaf people marry hearing people, and if these hearing people are fluent in Sign Language, the family language is likely to be Sign Language. These different family dynamics imply that children in such families will be raised in a Deaf environment, even if they are themselves hearing.

Moreover, Lane et al. (1996) assert that it is not the degree of audiological deafness that decides whether an individual is Deaf or not, but the degree of identification with the Deaf community. The deciding factor is usually ‘attitudinal deafness’ (Napier 2002). Hearing CODAs come from all ethnic, religious and economic backgrounds. The only common characteristic is having Deaf parents (Filer & Filer 2000). Children of Deaf adults may share and live unique life experiences. They experience Deafness as a typical part of their family life from childhood, and not as a shock or a foreign concept that they encounter in adulthood (Mand et al. 2009). Hoffmeister (2008) asserts that hearing children of Deaf parents are typically the successive generation in the Deaf community where Deaf people have hearing children when they marry. They represent a relatively invisible linguistic and cultural minority (Ladd 2003). Generally, they grow up as a part of the Deaf community and learn Sign Language as their first language (Bishop & Hicks 2005). Children of Deaf adults grow up in Deaf families, but not all CODAs grow up in a Deaf community (Hoffmeister 2008).

In as much as CODAs may be acculturated to Deaf ways within their families, their ability to hear creates uncertainty as to whether they are true inheritors of Deaf culture (Bishop & Hicks 2005; Preston 1994; Singleton & Tittle 2000). Initially, CODAs may not see themselves as hearing within their Deaf family and may only realise this when they are older (Bull 1998; Hoffmeister 1996). Understanding the life experiences of CODAs entails understanding that CODAs have an ongoing connection with the Deaf community, often sharing their views, and the experiences of suffering emotionally as a part of that community when Deafness is defined by some as a disability to be prevented where possible (Mand et al. 2009).

### Language brokering in Deaf families

Language brokering in Deaf-parented families arises from the fact that many Deaf adults may or may not have a reasonable ability to read and write spoken language and also may not be able to communicate adequately through spoken language (Hall & Guéry 2010). As a result, their children often act as language brokers between their Deaf parents and the hearing community (Hall & Guéry 2010). Language brokering in these families ranges from sporadic to regular, and CODAs are often forced to start language brokering from a very young age (Preston 1996).

According to Hall and Guéry (2010), CODAs start language brokering much earlier than the other children who may act as language brokers in families, for example, where parents may be immigrants. CODAs usually become language brokers because of the difficulties in interactions which may be owing to the fact that hearing family members often do not share the same language of communication as the Deaf parents, making access to social interactions difficult (Henderson & Hendershott 1991). As a result, in the presence of extended family or the hearing society at large, interactions may be affected as Deaf parents may not be able to communicate effectively, which will then necessitate that CODAs act as language brokers. Preston (1994) states that the oldest daughter often serves as the interpreter, even if she has an older brother and further asserts that female participants who did not consider themselves fluent in Sign Language still took on the interpreting responsibility. As a result, women were also more likely to become professional Sign Language interpreters than men, and men more likely to have poorly developed Sign Language skills in relation to female siblings (Preston 1996).

Some CODAs’ responsibility for handling family communication and the possible exposure to inappropriate context may create unwanted pressure and burdens which they are too young to resist or negotiate, and in most cases, the children may become emotionally involved in these interactions (Preston 1994; Singleton & Tittle 2000). However, despite some benefits, this interpreter role may place undue pressure on the hearing children of Deaf parents. It is suggested that hearing children of Deaf parents who act as language brokers may find themselves in a situation known as ‘role reversal’, which is a situation where a child feels responsible for the parents and the parents expect the child to be responsible for them (Buchino 1993).

Literature suggests that in some cases parents are aware of their reliance on their hearing children (Mallory et al. 1992; Torres 2003). Consequently, some parents opt not to use Sign Language with their children in order to prevent the possible overreliance on their children who are serving as interpreters (Jones, Strom & Daniels 1989). Furthermore, Morales and Hanson (2005) assert that children who served as language brokers also attempted to protect their parents from negative comments or embarrassment while interpreting. In an attempt to protect their Deaf parents, children who act as language brokers may not interpret insensitive remarks made by a hearing person about the Deaf parent as the hearing person may assume that all the family members are Deaf because they are using Sign Language to communicate. Also, within a confrontation between Deaf parents and hearing people, to avoid escalating the situation, CODAs may not interpret all of the parents’ angry statements or those of the hearing people (Filer & Filer 2000). It is clear that such situations present a challenge for CODAs, as they may find themselves caught between two worlds: one of the hearing community and one of the Deaf community.

Notwithstanding these challenges, some authors have highlighted that there are advantages for performing the roles of language and cultural brokers, as hearing children of Deaf parents gain valuable information about the adult world that might assist them in their own development. Furthermore, they also have an opportunity to develop a close relationship with their parents (Filer & Filer 2000). In addition, Preston (1994) asserts that hearing children of Deaf parents felt that their family experiences developed and encouraged their ability to empathise with others. An added bonus is that CODAs ‘enjoy a command of the languages and the cultural knowledge of two worlds’ and they benefit from that experience (Lane et al. 1996:171). Singleton and Tittle (2000) suggest that if the role of the parent is clear and the interpreting is kept to appropriate contexts, the added responsibility of interpreting can result in maturity, independence and an opportunity to have rich experiences. These authors claim that children who learn to navigate and explore the hearing world independently ‘develop positive attributes such as adaptiveness, resourcefulness, curiosity and “worldliness”’ (p. 228).

Not much is known about hearing children of Deaf parents in South Africa. A review of the existing literature into the patterns of language brokering in Deaf parents in a South African context yielded few results. For instance, locally, the Sowetan Newspaper (16 October 2012) published an article about a 3-year-old girl, Sfundo, who is the communication link within her family as well as between her family and the outside world. The article illustrates how children take up the essential role of interpreting at a very young age and shows how a 3-year-old girl acts as indispensable ‘ears’ for her Deaf parents. But when the parents are not at home, communication becomes harder and they have to rely on written notes to communicate. This article echoes the observations made by authors like Preston (1994) who notes that hearing children of Deaf parents start interpreting very early in life, shouldering responsibilities beyond their age. This 3-year-old girl is already interpreting for her family at a very young age. This is often the reality that many Deaf-parented families face.

As mentioned earlier, there is a dearth of knowledge regarding the experiences of CODAs in South Africa, which solicits the following questions: do we know enough about CODAs? Are CODAs not significant enough to demand the attention of professionals and researchers? The available data on the CODAs’ experiences from the United States and other contexts may not be applied easily into the South African context because of both linguistic and cultural diversity. Most studies on CODAs refer to CODAs as being bicultural and bilingual. This may not be the case in South Africa as some CODAs may view themselves as being both multicultural and multilingual.

The above factors necessitated a study of this nature with the aim to explore the experiences of CODAs within the specific context of Gauteng in South Africa in order to contribute to the gap in local knowledge. More specifically, this study intends to provide these adult children an opportunity to share and voice their experiences of being language brokers in their families. Authors internationally have shed some insights into the experiences of hearing children growing up in Deaf families; however, their insight may not be readily applied to the South African population, and hence the need for this current study.

### Objective

To explore the influence of CODAs’ gender and birth order on language brokering in the culturally Deaf family.

## Ethical consideration

Before commencing the study, approval was obtained from the University of the Witwatersrand’s Human Research Ethics committee (non-medical) (Protocol number: H110922). Furthermore, ethical aspects such as confidentiality and rights to withdraw from the study were considered. Anonymity, however, was not guaranteed as snowball sampling was utilised in this study.

## Method

### Research design

The goal for this study was to gain an insight into the CODA phenomenon in a South African context as experienced by CODAs, especially in relation to the influence of gender and birth order on language brokering in the family. Therefore, this study adopted a qualitative research design to describe the lived experiences of a sample of CODAs. A qualitative research allows for a ‘naturalistic approach that seeks to understand phenomena in context-specific settings, such as real world setting, where the researcher does not attempt to manipulate the phenomenon of interest’ (Patton 2005:39). More specifically, an interpretive phenomenological design was adopted to accurately capture the participants’ experiences and give them a voice to express these experiences (Larkin, Watts & Clifton 2006). This approach allowed for in-depth descriptions and understanding of the participants’ lived experiences as told from their perspectives (Babbie 2011). Therefore, the participants’ own words were used to express their experiences. The experiences generated rich, detailed and valid process information that contributed to an in-depth understanding of their context.

#### Procedure

Consent forms were formulated for the CODAs to participate in the study and for the interviews to be recorded digitally. The consent forms were written in English, highlighting the aims and the nature of the study. Also the participants were informed that participation was voluntary and that refusal to participate in or the decision to withdraw from the study carried no negative consequences. It was highlighted that anonymity was not guaranteed as this study relied on snowball sampling to obtain participants for the study. Participants were made aware that all information provided to the researcher would be kept confidential.

For inclusion in the study, participants had to meet the following criteria: must be CODAs between the ages of 18 and 40 years, would have been raised by their biological parent(s) as there may be different dynamics if the participants were raised by their extended family members and should be residents of Gauteng Province, an urban and, arguably, resourced province of South Africa.

#### Sample size and sampling strategy

A sample size of 10 hearing adult children of Deaf parents was obtained and interviewed for the study. The researcher predefined adult children of Deaf parents as a focus of this study. Therefore, the sampling strategy that was employed in this study was purposive sampling because it is a type of non-probability sampling which allowed the researcher to collect a sample from a population that met the inclusion criteria and was accessible to the researcher (Burns & Grove 2009). In conjunction with purposive sampling, the researcher also used snowball sampling to identify some of the participants for the study.

A snowball technique was necessary as this research sought to study a hidden population, for whom satisfactory lists and sampling frames are not readily available (Sadler et al. 2010). Snowball sampling method is defined as a sample design in which participants approach other people who meet the inclusion criteria defined by the researcher and request them to participate in the study. The technique enabled participants to put the researcher in touch with other possible participants (Sadler et al. 2010). Snowball sampling takes advantage of the social networks of identified participants to provide the researcher with an ever-expanding set of potential participants, allowing a series of referrals to be made within a circle of acquaintances (Robinson 2014). It is particularly effective in locating members of special populations where the focus of the study is on a sensitive issue (Sadler et al. 2010). As an audiologist working in the field, the principal researcher was familiar with CODAs. The CODAs known to the researcher were asked to act as gatekeepers and to approach other CODAs who could be interested in participating in the study and request them to participate. Potential participants were then put in contact with the researcher. In the event that CODAs were willing to participate, the gatekeepers were requested to grant permission for their CODAs’ contact details to be given to the researcher. This technique was an effective way to recruit participants for the study. From this sampling procedure, 10 CODAs agreed to participate in the study and their details are summarised in Table 1.

**TABLE 1:** Demographic profile of participants.

Participant	Gender	Age	Birth order	Race	Family dynamics
1	Female	22	First born	Black people	Both parents are Deaf. She has two Deaf siblings and one hearing sibling. She has a young daughter who is hearing. She interpreted for her parents as a child.
2	Female	30	First born	White people	Both parents are Deaf. All her siblings and children are hearing. She interpreted for her parents as a child.
3	Female	28	First born	White people	Both parents are Deaf. She has hearing siblings. As a child, she briefly interpreted for her family. The interpreter role was delegated to the younger siblings.
4	Female	24	Second born	White people	Both parents are Deaf. She has hearing siblings and is married to a hearing spouse. She interpreted for her parents as a child.
5	Male	22	Last born	White people	Both parents are Deaf. He has hearing siblings and interpreted briefly for his parents. Not fluent in South African Sign Language.
6	Female	40	Last born	White people	Both parents are Deaf. She has hearing siblings and children and is married to a CODA. She interpreted for her parents.
7	Female	26	Last born	Black people	Has a Deaf father and a hearing mother and sister. She interpreted for her father as a child. Older siblings did not sign fluently.
8	Female	30	Last born	White people	Both parents were Deaf. She has hearing siblings and interpreted for her parents as a child.
9	Female	35	First born	Black people	Both parents are Deaf. She has hearing siblings and once dated a Deaf person. She interpreted for her family as a child.
10	Male	26	Last born	Black people	Both parents are Deaf. He has hearing siblings and interpreted for his family.

### Semi-structured interviews

Questions for the semi-structured interviews were formulated by the researcher based on the available literature on CODAs in other countries. Furthermore, as per Kerlinger and Lee’s (2000) recommendation, similar questions were grouped together in order for cohesion and order. The interviews were conducted in a conversational manner, and the questions were not asked in any specific order; however, the first question was always used as the opening question. The other questions were asked in relation to the participant’s closing line.

#### Use of English language

Participants in this study were requested to indicate their language of preference for the interviews from the 12 South African languages, including South African Sign Language (SASL). All the participants preferred the use of English and as a result all the interviews were conducted primarily in English. Nevertheless, code switching was observed particularly in certain words and phrases. Code switching refers to a situation ‘wherein a person alternates between two languages within the same communicative event’ (Shulman & Capone 2010:361). This is often observed in individuals who are bilingual and in places where both languages are common in the environment (Owens 2012). Some participants occasionally used phrases from their home languages such as Zulu or Sotho to accurately capture or articulate their experiences. The interviewer is fluent in the participants’ home languages and so was able to maintain the conversation when such language switching occurred.

Overall the questions focused on five areas which were predefined by the researcher as being relevant to the study. The questions were based on the review of literature consulted for this study; the questions focused on the childhood experiences, interpreting experiences, occupational choices, support services and disability. More importantly, these questions were designed to answer or address the aims of the study and to answer the research questions posed. Where necessary, the participants were asked to elaborate and clarify. Generally, the questions were unambiguous and participants did not experience difficulties in answering the questions related to the gender and birth order as the portion of the study focused on that objective.

### Data analysis

The participants’ transcripts served as the raw data for this study and inductive thematic analysis was used as it allowed for the coding of data without trying to fit them into a pre-existing coding frame, or the researchers’ analytic preconceptions, and thereby allowing for themes to emerge from the data themselves (Braun & Clarke 2006). The themes were then analysed using the steps recommended by Creswell (2012). Representative verbatim quotations were used in the write-up of the study to support the findings.

### Trustworthiness

In order to deal with any bias or subjectivity in the handling and analysis of data, both as audiologists and non-CODAs, the authors had to acknowledge that ‘all research is subject to researcher bias’ (Morrow 2005:254). Therefore, reflexivity and bracketing were applied to guard against any bias from the authors. To achieve bracketing, a peer reviewer served as a mirror and assisted in reflecting on her responses to the interviews. Also, the authors made use of the ‘community of practice’ (Rossman & Rallis 2003:69) to share the process and the findings of the study with a group of colleagues in the department who are experienced researchers and are familiar with the current issues involving professionals working in the field of Deafness. After transcribing the interviews, the researcher realised the need to conduct member or participant checks through a focus group to ‘learn from the interviewee how well the researcher’s interpretations reflect the interviewee’s meaning’ (Morrow 2005:254). Furthermore, after transcribing the interviews, the researcher contacted some participants for more clarification where the researcher had misunderstood or sought extra information and such information was given.

### Pilot study

In order to ensure that the findings of this study yielded appropriate results, a pilot study was undertaken. The pilot study was conducted with one participant who was first to respond to the researcher’s request for participants for this study. The participant was a 22-year-old female who met the inclusion criteria of the current study. The interview was conducted in the researcher’s office as per the participant’s request. The interview was conducted in English and lasted for 45 min. The interview was audio recorded. The pilot study yielded no major changes to the interview guide; consequently, the data collected from the pilot study were included in the main study.

Generally, there is a common concern with the inclusion of the pilot study participants in the main study as those participants may already be exposed to an intervention and therefore may respond differently from other participants who were not included in the pilot sample. However, in some cases, ‘it may be impossible to exclude pilot-study participants because of small sample size’ (Kim 2011). This was the case with the current study as the sample size of the participants was very limited and it became necessary to include the data gathered during the pilot study. Also, the current study did not involve any intervention procedures or subsequent interviews. Kim (2011) further states that contamination is less of a concern in qualitative research as researchers often use some or all of their pilot data as part of the main study.

## Findings

The analysis of the interviews suggested that there were no formal rules when it came to assigning the role of interpreter in the family because CODAs reported that they had had to interpret for their parents at some point in time, regardless of the CODAs’ birth order or gender. For example, Participant 1 explained that, in her family, no one was formally asked to be the interpreter, ‘No one was given the role to interpret at home. We all interpret. Whoever is there interprets. No one was chosen to do it’. Furthermore, participants expressed that they would assume the role of interpreter out of necessity, without realising that this is what they are doing. Participant 7 made this point salient when she said:

‘You become an interpreter from the age of whatever without you realizing, because nobody understands your mother or your father and then now you have to go to hospitals with them, to clinics with them blah blah blah and they be like, “What is your father saying? What is he saying?”’

### Birth order

It appears as though the older children shifted the responsibility of interpreting to the younger siblings as exemplified by Participant 8, who said, ‘Well normally, it was the eldest in the house who would interpret, then as they moved out the next one would be the interpreter’. In this instance, it appears as though the shift in responsibility occurred when the older children moved out of the house and therefore passing the responsibility to the younger children. However, other participants indicated that the older children passed that responsibility on to younger ones, regardless of whether the older siblings were living in the house or not. This may be seen as normal progression where, when the older sibling leaves, the remaining ones take over the interpreter role. However, in this study, the results indicate that even when the older siblings were still at home, they still delegated this role to their younger siblings.

In this study, five participants were last-born children in their families and they all served as interpreters for their parents. These five participants mentioned that their older female siblings, who are first-born children, did not want to interpret and they believe that this was mainly because of personality differences. Participant 4 shared that her older sister, who is the first-born child in her family, preferred not to interpret for her family, as she explained: ‘My older sister was an introvert and she did not like interpreting as such. Eventually I did the most of the interpreting’. Similarly, Participant 7 also reported that her first-born sister did not interpret for her family, even though she thought that it would have been a role better suited to the older child:

‘I’m the last born at home and I have no idea how I ended up being an interpreter at home. But for some reason, with the CODAs I know, it’s usually the babies that tend to sign, or the second born or the third born or something. Not the first born. One would assume that the first born will take the responsibility. Not all CODAs. Like I said, the CODAs that I know, ja. But not all of them. It’s just like one or two CODAs that I know that are elderly at home would sign.’

This statement reinforces the notion that only a few of the older sibling CODAs are interpreters when they are living at home.

The results also revealed that younger children were expected to interpret on topics that were not age appropriate; however, because of older siblings delegating the interpreter role to younger ones, these younger children had to engage in difficult conversations. For example, Participant 8, the youngest child in the family, said:

‘The phone rang one time and it was my aunt saying that my grandfather has passed away. And you have to tell that to your parents. It’s awful. It’s awful telling your mom, ‘Hey your dad just died’. She just started crying and I didn’t know what to do. I just turned around and walked away. You know, what do you do?’

Participant 4, who has an older sibling, felt that she was expected to do things her older sibling may have been better suited to doing:

‘We were exposed to grown-up business at a young age because we are the mode of communication and having that responsibility already from a young age. Answering the door. Answering the phone. Speaking to people, querying things, communicating for your parents towards someone else. You immediately assume responsibility. You need to focus and try and explain what they are trying to say and not be a child, if you don’t understand, you just can’t go on with your life. You know you have this responsibility; otherwise, miscommunication can affect you and so on.’

These findings highlight that interpreting goes beyond passing on information. The interpreter role necessitated that children guarded against miscommunications, as this will have a bearing on how the parents understand what was communicated to them.

### Gender

Although the participants had said that there was no formal assignment of the interpreter role, they reported that female children tended to assume the role of interpreter more than the male children in their families. Some of the participants speculated that this might have been because of being shy of having Deaf parents or attracting attention to oneself when assuming the signing role, especially in public. One male participant in this study indicated that he is not fluent in SASL as he rarely signed for his parents. Three participants mentioned that they have male siblings but these male siblings did not want to interpret for their Deaf parents, so the female siblings took on that role. Participant 6 reported that her brother did not interpret for the family and, more specifically, it seemed to her that he was embarrassed about having Deaf parents:

‘My brother was never interested. My brother would run away very far, he’s not into it. He could sign very little, very limited. I wouldn’t classify my brother as a shy person but you know, we would walk in the street and he would tell my mother not to sign. He was shy of that aspect. I think he was shy of having Deaf parents. So he just never did it and it was never … it just became the females’ job.’

Participant 5, a male participant, reinforced this point when he said that he was constantly aware of the attention he was drawing from the hearing community and the embarrassment which accompanied it:

‘It’s almost like being ashamed of having Deaf parents in a hearing place. They, you know the way they speak sometimes. Their voicing is not a normal way of speaking. The noises (of disapproval from people around them) that you hear. Need to check for tension (in that situation). So it depends on the situation. That can also make you feel self-aware; ‘Oh people are looking at us’. That can also have an effect.’

Female participants expressed the view that their male siblings would relegate the role of interpreter to them and that this role was not necessarily their choice, but was a role they assumed out of a sense of duty. For instance, Participant 8 described how she and her siblings fought over who was going to interpret for their family because no one in her family wanted to interpret for the parents:

‘I don’t know. I remember that we used to fight about who is going to interpret. Like you didn’t want to. It wasn’t really something that you wanted to do. It was like ‘not again’ but you had to do it.’

Furthermore, female participants felt that because the interpreting role is often assumed by the female CODAs, they found themselves having to discuss topics which they, as females, found difficult to interpret with their fathers. Participant 9 described a situation where she had to interpret about rape when she said:

‘I remember, when I was 10, I had to interpret ‘rape’, and I didn’t even know what rape was and because the news reader was also not explicit, I just spelled it and my father explained what rape was and for me it was such a shock.’

In other cases, female interpreter roles may have been better matched, as daughters, and as females, to interpret for their mothers, even if the topics were still difficult to discuss. Participant 9 shared an event where she had to explain to her mom about hysterectomy, even though she found it difficult: ‘I have pictures (mental images) of my mother having a hysterectomy and I had to interpret when the doctor came in afterwards’.

In this study, interpreting was voiced as one of the most sensitive and complex tasks for CODAs. All the CODAs in this study stated that they have at some point interpreted for their parents, even if they had not wanted to or not chosen to do so. In most cases, the CODAs’ reluctance to sign for their parents may have been because of the sensitive nature of the content and the situations they found themselves in. Therefore, it seems as though gender and birth order considerations play out in CODAs communication experience. The discussion below provides some insights into these findings.

## Discussion

The objective of this study was to explore the influence of CODAs’ gender and birth order on language brokering in the culturally Deaf family. From this study, it was apparent that all the participants interpreted for their Deaf parents, even those who may have not wanted to do so because of the nature of the content, being shy and embarrassed of having Deaf parents or not wanting to draw attention from the hearing community. More specifically, in this study, the role of a language broker was delegated to the youngest child, which is different from previous studies in the field where, for example, Buchino (1993) and Preston (1994) found that the oldest child interpreted for the parents. The difference in results of these studies conducted in the 1990s and the current study show that, although the research interest in CODAs may have waned, it still remains vital to conduct ongoing research in the area because it is apparent that there are changes in the pattern of CODA interpreting roles across time and in different contexts. However, the findings of this study indicate that younger siblings may be assigned the role of taking care of the communication needs of the parents when the older siblings move out of home. This shift in responsibility may explain the difference between the studies conducted in the USA (Buchino 1993; Preston 1994) and this study conducted in South Africa. This accentuates the need for ongoing and context-based research.

This study revealed that female CODAs are more likely to interpret for their families and male children are less likely to do so, which appears to correlate with other studies (Buriel et al. 1998; Love 2003; Mallory et al. 1992; Preston 1994). Also according Buriel et al. (1998) and Love (2003), female children are more likely to act as interpreters than male children. Preston (1996) rationalises that females often assume the interpreter role as interpreting entails behaviours and skills often culturally ascribed to women such as helping, connecting, mediating, bridging and caretaking. The female participants in this study indicated that they embraced this role out of necessity as they did not have access to interpreting services.

Moreover, the participants in this study indicated that they have to interpret in some situations where they feel they are not developmentally or emotionally ready or in situations which they feel are better suited for older siblings or for siblings of another gender. Furthermore, over and above interpreting, the CODAs in this study highlighted the importance of maintaining and facilitating communication so that there is no communication breakdown between the parties involved. Therefore, they had to facilitate communication and not simply interpret or convey what was being said. DeMent and Buriel (1999) and Tse (1995) stated that the role of interpreters is to facilitate communication between two linguistically and/or culturally different communities, and not only conveying information, which the CODAs in this study seemed to have done. This added responsibility may place CODAs under pressure to ensure that communication is successful, even in situations that they feel are not ideal.

## Recommendations of the study

Cognisant of the contextual constraints and resource limitations in South Africa for people who are Deaf, the recommendations may seem lofty and aspirational but are necessary in terms of planning and resource allocation. This study highlights the pressure placed on CODAs to interpret for their Deaf parents, thereby highlighting the need for official and non-family member interpreters for Deaf families. The availability of interpreters will alleviate the pressure placed on CODAs, who currently find themselves interpreting in situations that are not ideal emotionally, developmentally and psychosocially. In order to facilitate the availability of professional interpreters for Deaf people, there is the need for formalisation of interpreting services for Deaf people in South Africa rather than the reliance on CODAs to interpret. However, as mentioned earlier, this aspiration is idealistic within the resource-constrained context of South Africa and points to the need of exploring greater budget allocations for interpreting services, not as a nice-to-have but as an essential component of service provision and human rights. For example, the allocations made for interpreters of spoken languages such as Afrikaans, isiZulu, isiXhosa and the other official languages need to account for the needs of people whose first language is SASL. These alternatives could offer support for CODAs while, admittedly, not resolving their challenges because it creates a space for freedom from the imposition upon them, especially for young females. The recommendation by the Pan South African Language Board (PanSALB) that SASL should be recognised as an official language may go a long way towards legitimising and formalising language services for people who are Deaf, which, in turn, may have a positive spin-off on CODAs by alleviating them of their added, and often onerous, responsibility. Therefore, this recommendation by PanSALB is encouraged as this endorsement and recognition by government would have to provide for the training of interpreters and would also have to create opportunities for families from lower socio-economic backgrounds to access interpreter services. This right to interpreting services would then be entrenched in the people’s constitutional rights.

Moreover, in terms of family dynamics, it is recommended that strategies should be made available by professionals who interact with CODAs to provide strategic support. One such strategic support mechanism could be the use of the Family Systems Perspective (FSP) as discussed by Jackson and Turnbull (2004). The FSP addresses the impact of Deafness on the quality of life in the family. It identifies four crucial aspects of any family: family interactions, family resources, parenting and support for the child. Also, the FSP ‘acknowledges the mutual impact of each member’s strengths and needs and recognises the importance of addressing issues related to family life’ (Jackson & Turnbull 2004:15):

Because the deaf person is a component of the family system, the deafness belongs not just to the affected individual but to the entire family. Accepting this perspective makes it necessary for the family to seek ways to recognize itself so that all the components in the family system can participate, contribute, and draw on the family’s resources equally. (Henderson & Hendershott 1991:325)

Poston et al. (2003) state that implementing the model of family quality of life assists in embracing the overall degree to which the needs of each family member are met, the degree to which they enjoy family interactions and the degree to which they are able to participate in activities that are important to them as a family. Families and service providers such as audiologists, psychologists, doctors and policymakers may need to evaluate working ‘hand-in hand’ to manage any barriers families may encounter. Service providers may assist in arranging interpreting services, obtaining close captioning and securing funding for interpreting services at community events and activities (Jackson & Turnbull 2004).

## Limitations of the study

This current study sampled participants from Gauteng, a more urban and economically active province in South Africa; therefore, the participants’ experiences cannot be seen to be representative of all hearing children born to Deaf parents across South Africa, as the experiences of hearing children residing in other provinces may differ from the experiences of CODAs interviewed in this study.

Also in this study, the participants were asked to recount past events and childhood experiences and there is a possibility that recounting the experiences of growing up in Deaf-parented families may result in restructured or altered memories where participants may not accurately recall the events as they occurred. However, some researchers have employed similar methods to collect data for their studies (Christodoulou et al. 2009; Preston 1994; 1996).

A possible limitation may be the use of references which are not very recent. However, these references point to the hiatus in the research on CODAs and for updated research in the area as it is an area of importance.

## Further research

As not much is known about CODAs in South Africa and the current study explored the experiences of a cohort of CODAs in Gauteng only, it may be beneficial to conduct a similar study in other provinces of South Africa. Also, a larger sample of CODAs may add more richness and more information on the experiences of CODAs in all the provinces across South Africa.

Notwithstanding that the first author and interviewer is herself black and female and her attempts to recruit participants from a range of different cultures and races, the majority of the participants in this study were white and females. Conducting a similar study focusing on the experiences of black CODAs in South Africa to capture the possible similarities and differences between the white and the black CODAs may add to understanding the interpreter role within a culturally diverse South Africa.

## References

[CIT0001] Babbie E., 2011, , 5th edn., Wadsworth, Cengage Learning, Belmont, CA.

[CIT0002] Baker K.A., 2011, , viewed n.d., from 2011 http://southafrica.to/history/Apartheid/apartheid.htm

[CIT0003] Barratt J.F., 2007, ‘The experience of caring for a child with cerebral palsy in Tonga, Mpumalanga: Caregiver’s stories’, MA dissertation, Speech Pathology and Audiology, University of the Witwatersrand.

[CIT0004] Bishop M. & Hicks S., 2005, ‘Orange eyes: Bimodal bilingualism in hearing adults from deaf families’, 5, 188–230. https://doi.org/10.1353/sls.2005.0001

[CIT0005] Braun V. & Clarke V., 2006, ‘Using thematic analysis in psychology’, 3(2), 77–101. https://doi.org/10.1191/1478088706qp063oa

[CIT0006] Buchino M.A., 1993, ‘Perceptions of the oldest hearing child of deaf parents: On interpreting, communication, feeling and role reversal’, 138(1), 40–45. https://doi.org/10.1353/aad.2012.0598 10.1353/aad.2012.0598 8484351

[CIT0007] Bull T., 1998, , Deaf Family Research Press, Alexandria, VA.

[CIT0008] Buriel R., Perez W., DeMent T.L., Chavez D.V. & Moren V.R., 1998, ‘The relationship of language brokering to academic performance, biculturalism, and self-efficacy among Latino adolescents’, 20(3), 283–297. https://doi.org/10.1177/07399863980203001

[CIT0009] Burns N. & Grove S.K., 2009, , Saunders Elsevier, St. Louis, MO.

[CIT0010] Christodoulou D., Hadjidemetri E., Konidari M. & Nicolaou N., 2009, ‘The experiences of Cypriot hearing adults with deaf parents in family, school, and society’, 14(4), 486–502. https://doi.org/10.1093/deafed/enp011 10.1093/deafed/enp011 19502571

[CIT0011] Clark K., 2003, , viewed 23 July 2017, from http://www.lifeprint.com/asl101/pages-layout/coda.htm

[CIT0012] Creswell J.W., 2012, , Pearson Education, Inc., Upper Saddle River, NJ.

[CIT0013] DeMent T. & Buriel R.W., 1999, ‘Children as cultural brokers: Recollections of college students’, paper presented at the SPSSI Conference on Immigrants and Immigration, Toronto, Canada, August 1999.

[CIT0014] Filer R.D. & Filer P.A., 2000, ‘Practical considerations for counselors working with hearing children of Deaf parents’, 78, 38–43. https://doi.org/10.1002/j.1556-6676.2000.tb02558.x

[CIT0015] Hall N. & Guéry F., 2010, , mediAzioni 10, viewed n.d., from http://mediazioni.sitlec.unibo.it

[CIT0016] Henderson D. & Hendershott A., 1991, ‘ASL and the family system’, 136(4), 325–329. https://doi.org/10.1353/aad.2012.0546 10.1353/aad.2012.0546 1750372

[CIT0017] Hoffmeister R., 1996, ‘A piece of the puzzle. ASL and reading comprehension in deaf children’, paper presented at the Theoretical Issues in Sign Language Research, Montreal, Quebec, Canada, September.

[CIT0018] Hoffmeister R., 2008, , University of Minnesota Press, Minneapolis, MN.

[CIT0019] Jackson C.W. & Turnbull A.P., 2004, ‘Impact of deafness on family life: A review of the literature’, 24(1), 15–29. https://doi.org/10.1177/02711214040240010201

[CIT0020] Jones E., Strom R. & Daniels S., 1989, ‘Evaluating the success of deaf parents’, 134, 312–316. https://doi.org/10.1353/aad.2012.0511 10.1353/aad.2012.0511 2618919

[CIT0021] Kerlinger F.N. & Lee H.B., 2000, , 4th edn., Harcourt College Publishers, Fort Worth, TX.

[CIT0022] Kim Y., 2011, ‘The pilot study in qualitative inquiry’, 10(2), 190–206. https://doi.org/10.1177/1473325010362001

[CIT0023] Ladd P., 2003, , Multilingual Matters, Levedon, UK.

[CIT0024] Lane H., Hoffmeister R. & Bahan B., 1996, , Dawn Sign Press, San Diego, CA.

[CIT0025] Larkin M., Watts S. & Clifton E., 2006, ‘Giving voice and making sense in interpretative phenomenological analysis’, 3(2), 102–120. https://doi.org/10.1191/1478088706qp062oa

[CIT0026] Love J.A., 2003, April, ‘Language brokering, autonomy, parent-child bonding, and depression’, paper presented at the 2003 Conference of the Society on the Research of Child Development Miami.

[CIT0027] Lucas C. & Valli C., 1990, , Gallaudet University, Washington, DC.

[CIT0028] Mallory B.L., Schein J.D. & Zingle H.W., 1992, ‘Improving the validity of the PSNI in assessing the performance of deaf parents of hearing children’, 137, 14–21. https://doi.org/10.1353/aad.2012.0473 10.1353/aad.2012.0473 1605094

[CIT0029] Mand C., Duncan R.E., Gillam L., Collins V. & Delatycki M.B., 2009, ‘Genetic selection for deafness: The views of hearing children of deaf adults’, 35, 722–728. https://doi.org/10.1136/jme.2009.030429 10.1136/jme.2009.030429 19948926

[CIT0030] McIlroy G.W., 2008, ‘A narrative exploration of educational experiences on deaf identity’, Unpublished Master’s research report, University of the Witwatersrand, Johannesburg.

[CIT0031] Morales A. & Hanson W.E., 2005, ‘Language brokering: An integrative review of the literature’, 27(4), 471–503.

[CIT0032] Morrow S.L., 2005, ‘Quality and trustworthiness in qualitative research in counseling psychology’, 52(2), 250–260. https://doi.org/10.1037/0022-0167.52.2.250

[CIT0033] Murray J.B., Klinger L. & McKinnon C.C., 2007, ‘The deaf: An exploration of their participation in community life’, 27(3), 113–120.

[CIT0034] Napier J., 2002, ‘The D/deaf-H/hearing debate’, 2(2), 141–149. https://doi.org/10.1353/sls.2002.0006

[CIT0035] Owens R., 2012, , 8th edn., Pearson, Boston, MA.

[CIT0036] Patton M.Q., 2005, , John Wiley & Sons.

[CIT0037] Poston D., Park J., Turnbull A.P., Mannan H. & Marquis J., 2003, ‘Family quality of life outcomes: A qualitative inquiry launching a long-term research program’, 41(15), 313–328. https://doi.org/10.1352/0047-6765(2003)41<313:FQOLAQ>2.0.CO;2 10.1352/0047-6765(2003)41<313:FQOLAQ>2.0.CO;2 12962536

[CIT0038] Power D. & Leigh G., 2003, , Oxford University Press, London.

[CIT0039] Preston P., 1994, , Harvard University Press, Cambridge, MA.

[CIT0040] Preston P., 1995, ‘Mother father deaf: The heritage of difference’, 40(11), 1461–1467. https://doi.org/10.1016/0277-9536(94)00357-Y 10.1016/0277-9536(94)00357-y 7667651

[CIT0041] Preston P., 1996, ‘Chameleon voice: Interpreting for deaf parents’, 42(12), 1681–1690. https://doi.org/10.1016/0277-9536(95)00299-5 10.1016/0277-9536(95)00299-5 8783430

[CIT0042] Quigley S. & Paul P., 1990, , College Hill, San Diego, CA.

[CIT0043] Robinson O.C., 2014, ‘Sampling in interview-based qualitative research: A theoretical and practical guide’, 11, 25–41. https://doi.org/10.1080/14780887.2013.801543

[CIT0044] Rossman G.B. & Rallis S.F., 2003, , Sage, Thousand Oaks, CA.

[CIT0045] Sadler G., Lee H., Lim R. & Fullerton J., 2010, ‘Recruitment of hard-to-reach population subgroups via adaptations of the snowball sampling strategy’, 12, 369–374. https://doi.org/10.1111/j.1442-2018.2010.00541.x 10.1111/j.1442-2018.2010.00541.x PMC3222300 20727089

[CIT0046] Selzer M., 2010, ‘South African Sign Language used in Parliament: Is there a need for standardisation?’ MA dissertation., Stellenbosch University, Cape Town.

[CIT0047] Shulman B. & Capone N., 2010, , 2nd edn., Jones and Bartlett Publishers, Burlington.

[CIT0048] Singleton J.L. & Tittle M.D., 2000, ‘Deaf parents and their hearing children’, 5, 221–236. https://doi.org/10.1093/deafed/5.3.221 10.1093/deafed/5.3.221 15454502

[CIT0049] Siple L.A., 1994, ‘Cultural patterns of deaf people’, 18(3), 345–367. https://doi.org/10.1016/0147-1767(94)90037-X

[CIT0050] Storbeck C., 2010, ‘Postgraduate study for deaf South Africans’, 155(4), 502–503. https://doi.org/10.1353/aad.2010.0032

[CIT0051] Torres M.T.W., 2003, ‘A phenomenological study of the parenting experiences of deaf adults’, Doctoral dissertation, Our Lady of the Lake University.

[CIT0052] Tse L., 1995, ‘Language brokering among Latino adolescents: Prevalence, attitudes, and school performance’, 17(2), 180–193. https://doi.org/10.1177/07399863950172003

